# SMARCA4‐deficient lung tumour that presented with haemoptysis and progressed rapidly

**DOI:** 10.1002/rcr2.656

**Published:** 2020-08-31

**Authors:** Mari Inoue, Tatsuji Enomoto, Masashi Kawamoto, Naoto Mikami, Hidehiko Kuribayashi, Noriyuki Saeki

**Affiliations:** ^1^ Department of Respiratory Disease Center Ofuna Chuo Hospital Kamakura Japan; ^2^ Department of Diagnostic Pathology Teikyo University Hospital, Mizonokuchi Kawasaki Japan

**Keywords:** Haemoptysis, lung, rhabdoid, sarcomatoid, SMARCA4

## Abstract

The case of a heavy ex‐smoking man in his early 70s who presented with haemoptysis and died following rapid progression is presented. The tumour excised by surgery was mostly composed of monotonous large rhabdoid cells showing prominent nucleoli and eosinophilic cytoplasm. On immunohistochemistry with SMARCA4 (BRG‐1), the tumour cells showed significant loss of expression. The tumour was diagnosed as a SMARCA4‐deficient thoracic sarcoma. This is a disease that progresses rapidly and has a poor prognosis. However, the search for specific treatments using synthetic lethality is underway. Clinical and pathological characteristics can be identified with examination of more cases, and when the tumour is suspected, it is necessary to actively perform immunohistochemical examination.

## Graphical abstract

We present the case of a heavy ex‐smoking man in his early 70s who presented with haemoptysis and died following rapid progression. The excised tumour was diagnosed as a SMARCA4‐deficient thoracic sarcoma. This is a disease that progresses rapidly and has a poor prognosis.

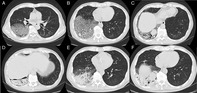

## Introduction

SMARCA4‐deficient tumours have been recognized in recent years [1, 2]. It is said that the prognosis of patients with these tumours is extremely poor, and it is necessary to examine more cases and clarify their clinical characteristics [3, 4, 5]. A case of a patient with a SMARCA4‐deficient lung tumour who presented with bloody sputum and then died following rapid progression is presented.

## Case Report

A man in his early 70s with an ex‐smoking history (63 pack‐years) suddenly developed bloody sputum. Even though the episode of bloody sputum continued, his general condition was very good, and he went mountain climbing. Nine days after the first episode of bloody sputum, he visited an otolaryngologist who found no apparent abnormality. However, his bloody sputum did not improve, but got worse, and he developed haemoptysis. He visited a physician who diagnosed pneumonia, and he was referred to our hospital two weeks after the initial symptom.

His consciousness was clear, temperature 36.4°C, blood pressure 123/71 mmHg, pulse 71/min, and oxygen saturation was 95% (room air). The physical examination was normal except for decreased right‐sided breath sounds. Blood tests showed decreased haemoglobin (10.7 g/dL) and increased C‐reactive protein (10.75 mg/dL). There were no other abnormalities, including tumour markers.

**Figure 1:**
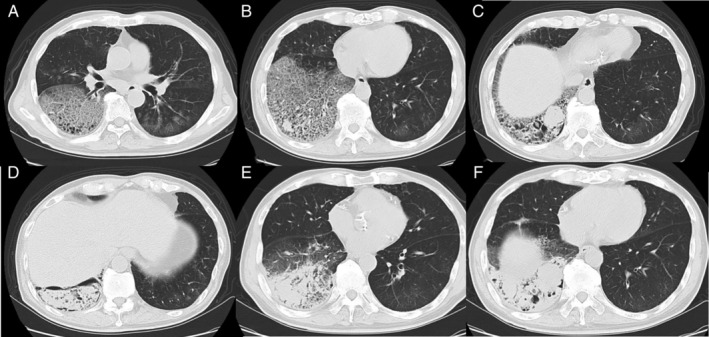
Chest computed tomography (CT) on the admission day shows widespread ground‐glass opacity with intralobular septal thickening in the right lower lobe (A), irregular surface masses in the right S9 (15 mm) (B) and right S10 (33 mm) (C), a relatively large cavity‐like lesion including a solid substance inside in the right basal area (D), and spotty ground‐glass opacity in the whole left field (A–D). CT after 18 days of admission (E, F) shows that, although the extent of ground‐glass opacity has decreased, the masses of right S9 and S10 have increased, and the surrounding consolidation has become stronger, accompanied by a decrease in structure.

Chest computed tomography (CT) showed widespread ground‐glass opacity with interlobular thickening in the right lower lobe (Fig. 1A). Two marginal irregular masses, 15 mm in the right S9 (Fig. 1B) and 33 mm in the right S10 (Fig. 1C), were observed. Even lower on the right side, a relatively large cavity‐like lesion in which there was substantial material inside (Fig. 1D) was seen. There was notable patchy ground‐glass opacity in the left lung as well. In addition, the hilar and subtracheal lymph nodes were slightly enlarged.

On the admission day, he was diagnosed with pneumonia with airway haemorrhage, and haemostasis and antibiotics were started. Bronchoscopy was performed, and blood was seen to be attached to the trachea and all bronchi. Although right B10 was considered to be the bronchus causing the bleeding, no tumours or vascular lesions were observed. It was decided that transbronchial biopsy would be unsafe, and bronchial washings from the right B10 were collected. No significant findings were obtained on cytological and bacterial examinations of the lavage fluid.

The patient was then treated with various antibacterial and antifungal agents, as well as blood products for systemic management, but he did not improve. CT after 18 days of admission (Fig. 1E, F) showed that, although the extent of ground‐glass opacity was decreased, the masses in right S9 and S10 were increased, and the surrounding consolidation had become denser with volume loss. A pleural effusion had appeared. Some nodules of suspected pleural seeding were also seen, and the hilar and subtracheal lymph nodes were further enlarged. On the systemic CT survey, no distant metastases were observed.

Twenty days after admission, right lower lobectomy and lymph node dissection were performed. The surgical findings showed that a bloody pleural effusion filled the thoracic cavity, and the right lower lobe was changed in colour to dark purple and hard. White nodules were observed on the pleural surface.

On gross examination of the tumour excised by surgery, it was a 70 × 45 × 90 mm^3^, coarse, continuous multinodular mass with a milky white tone on the cut surface. Blood was noticeable in the surrounding area, especially in the distal side of the tumour. Histological examination did not provide a final diagnosis, as it just mentioned large cell carcinoma or thoracic sarcoma, such as rhabdomyosarcoma.

The patient's immediate post‐operative course was relatively good, but the residual tumours increased rapidly, and respiratory failure progressed. Best supportive care was carried out according to the wishes of the patient and family. The patient died one month post‐operatively, 1.5 months after hospitalization.

**Figure 2:**
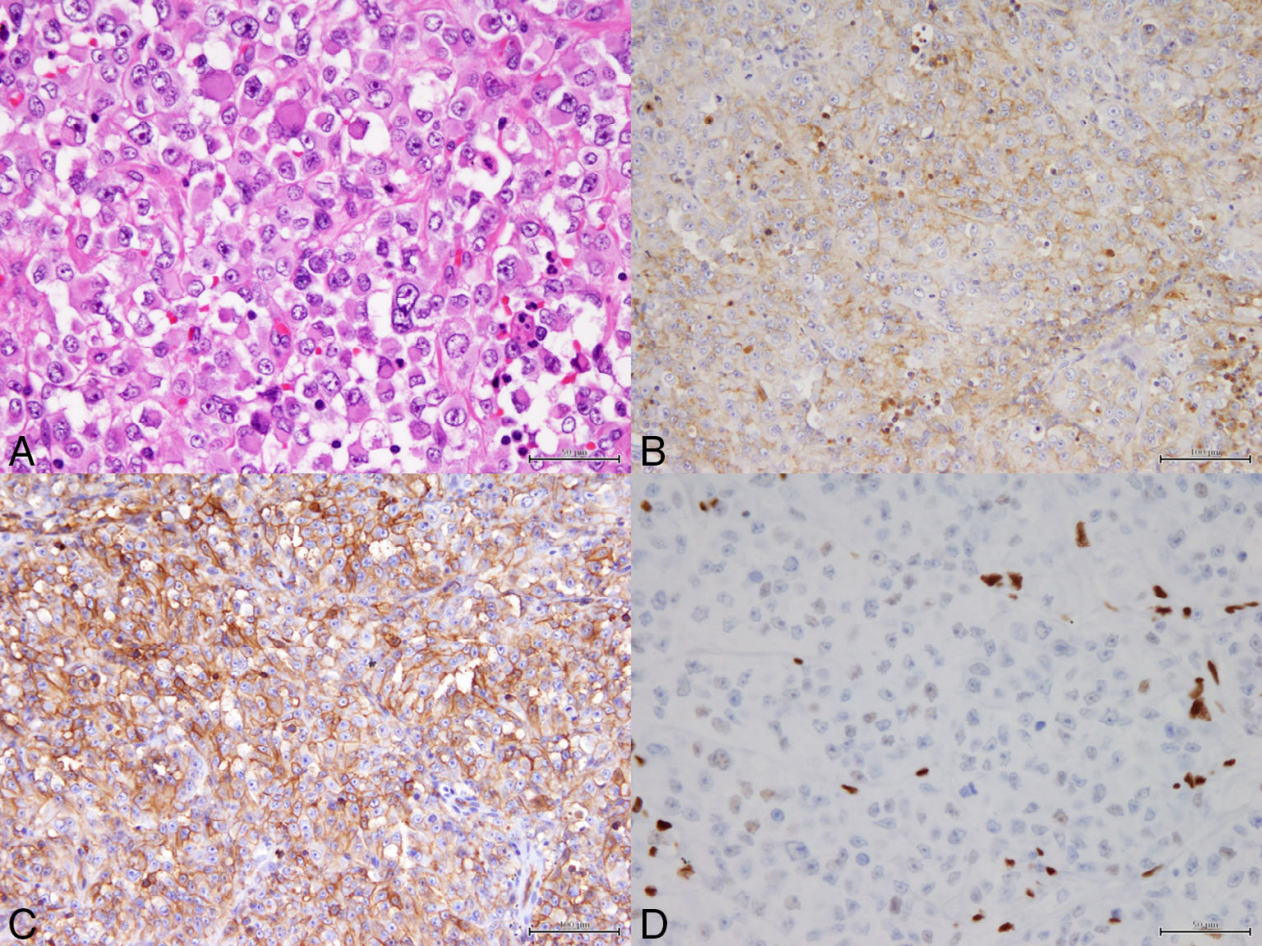
Histological examination. Resected tumour is almost composed of monotonous large rhabdoid cells showing prominent nucleoli and eosinophilic cytoplasm (A: haematoxylin and eosin (H&E) staining). Immunohistochemistry shows positive for EMA (B)and CD34 (C), but diffuse severe reduction of SMARCA4 is noted (D).

Later on, the final pathological diagnosis was as follows. The resected tumour was mostly composed of monotonous large rhabdoid cells showing prominent nucleoli and eosinophilic cytoplasm (Fig. 2). On immunohistochemistry with SMARCA4 (BRG‐1), the tumour cells showed significant loss of expression. Other immunostaining showed AE1/AE3 (−), vimentin (+), EMA (+), TTF‐1 (−), p40 (−), Calretinin (−), Claudin‐4 (−), CD34 (+), HMB45 (−), and desmin (−). Myogenin could not be determined due to the background staining. From the above, the tumour was diagnosed as a SMARCA4‐deficient thoracic sarcoma.

## Discussion

SMARCA4‐deficient tumours have been recognized in recent years, but they have not been established in both sarcomas and carcinomas [1, 2].

It has been reported that the clinical characteristics of patients with SMARCA4‐deficient tumours are young onset (median age: 39–48, range: 27–90 years), predominantly men (90.0–91.6%), heavy smokers (70% are 20 pack‐years or more, or median 18.5 pack‐years, range: 5–60 pack‐years) with emphysematous changes in the lungs. Furthermore, they progress rapidly without brain metastasis, often with peritoneal metastasis, and the prognosis is extremely poor, with a median overall survival of six to seven months [3, 4].

Although the present patient was relatively old, in his early 70s, the other characteristics, a heavy‐smoking (63 pack‐years) man with emphysematous changes in the lungs, rapid progression, and a poor prognosis, match the previous reports. In this case, it took only two months from onset to death.

Many of the main complaints of previously reported cases were pain, such as back pain. Within the scope of our literature search, there were no reports of uncontrollable haemoptysis as the chief complaint.

The morphological findings of reported cases of SMARCA4‐deficient thoracic sarcomas showed diffuse sheets of mildly dyscohesive, relatively monotonous, and undifferentiated epithelioid cells with prominent nucleoli [5]. Varying degrees of rhabdoid cells were also reported [3, 4]. In this regard, pathologists should be aware of this sarcoma when a tumour is undifferentiated, especially when reminiscent of rhabdoid‐type sarcoma. In the present case, the tumour consisted mostly of monotonous rhabdoid cells except for necrosis, so “large cell carcinoma with rhabdoid phenotype of the lung (LCCRP) (former classification of World Health Organization (WHO) 2004)” was the first diagnosis to come to mind. In the latest WHO classification, LCCRP may be included in the category of “large cell carcinoma with null or unclear immunohistochemical features,” but SMARCA4‐deficient thoracic sarcoma may be one option or will be replaced by LCCRP. Moreover, differential diagnosis between sarcomatoid carcinoma (pleomorphic carcinoma) and true sarcoma is difficult in the absence of an apparent non‐small cell carcinoma component, as in the present case. Rekhtman et al. suggested “SMARCA4‐deficient thoracic sarcomatoid tumours” for thoracic tumours with SMARCA4 deficiency and undifferentiated round cell and/or rhabdoid morphology [2].

The SMARCA4 complex belongs to the SWI/SNF family and is one of the chromatin control genes. Inactivated gene products themselves are not therapeutic targets. Therefore, it has been reported that SMARCA4/BRG‐1‐deficient tumours can be treated by molecular targeted therapy, and that cell death can be caused by inhibition of the functional paralog gene SMARCA2/BRM due to synthetic lethality [6].

In the future, diagnosis may be directly linked to treatment, and further clinical and pathological characteristics as more cases are examined will need to be elucidated.

### Disclosure Statement

Appropriate written informed consent was obtained for publication of this case report and accompanying images.
